# Early Experience with the New XEN63 Implant in Primary Open-Angle Glaucoma Patients: Clinical Outcomes

**DOI:** 10.3390/jcm10081628

**Published:** 2021-04-12

**Authors:** Antonio Maria Fea, Martina Menchini, Alessandro Rossi, Chiara Posarelli, Lorenza Malinverni, Michele Figus

**Affiliations:** 1 Struttura Complessa Oculistica, Città Della Salute e Della Scienza di Torino, Dipartimento di Scienze Chirurgiche-Università Degli Studi di Torino, 10126 Torino, Italy; alessandro.rossi012309@gmail.com (A.R.); lorenza.malinverni@unito.it (L.M.); 2 Department of Surgical, Medical and Molecular Pathology and Critical Care Medicine, University of Pisa, 56126 Pisa, Italy; martina.mmenchini@gmail.com (M.M.); chiara.posarelli@med.unipi.it (C.P.); michele.figus@unipi.it (M.F.)

**Keywords:** glaucoma, open-angle glaucoma, XEN, minimally invasive glaucoma surgery, intraocular pressure, glaucoma surgery

## Abstract

The new XEN63 implant is a minimally invasive glaucoma surgery device with limited experience in real life. This retrospective study included open-angle glaucoma patients who underwent XEN63 implant, either alone or in combination with cataract surgery. Primary endpoints were the intraocular pressure (IOP) at month 3 and the incidence of serious adverse events. Twenty-three eyes of 23 patients were included. Mean age was 67.8 ± 15.3 years and 15 (65.2%) were women. Mean IOP was significantly lowered from 27.0 ± 7.8 mmHg at baseline to 12.2 ± 3.4 mmHg at month 3 (*p* < 0.0001). Mean IOP lowering was 40.8 ± 23.5%, with 14 (60.9%) and 16 (69.6%) eyes achieving an IOP lowering ≥30% and ≥20% without hypotensive medication, respectively. The number of hypotensive medications (NHM) was significantly reduced from 2.27 ± 0.94 drugs at baseline to 0.09 ± 0.42 drugs at month 3, *p* < 0.0001. Four (17.4%) eyes had hypotony (IOP ≤ 6 mmHg) at postoperative day one, which was successfully resolved without sequelae. Four (17.4%) eyes had choroidal detachment (3 at day 7 and 1 at day 15), which was successfully resolved with medical treatment, at the month 1 visit. Three (13.0%) eyes required needling (mean time for needling 35.6 ± 9.7 days). XEN63 significantly lowered IOP and reduced the NHM, with a good short-term safety profile.

## 1. Introduction

The term glaucoma covers a wide range of multifactorial, chronic, and progressive optic neuropathies, characterized by progressive loss of retinal ganglion cells and subsequent visual field defects [1].

Glaucoma is a leading cause of irreversible blindness, and is estimated to affect over 111 million people worldwide by 2040 [2].

The main goal of glaucoma treatment is to slow the progression of the disease and to preserve, as much as possible, the patient quality of life.

Up to now, decreasing intraocular pressure (IOP) has been the only proven method to treat glaucoma [3]. To do so, different treatment strategies, such as medical therapy, laser, and surgery are currently available.

Although topical hypotensive medication is usually the first treatment approach, many patients do not achieve adequate glaucoma control due to different causes, including poor adherence, side effects, or lack of maintained efficacy [4,5].

Despite trabeculectomy being considered the gold standard in glaucoma surgery, due mainly to its well-established efficacy in terms of lowering IOP [6], it may lead to potential vision-threatening complications [7].

Minimally invasive glaucoma surgery (MIGS) devices have been developed as a safer and less traumatic means of lowering IOP in patients with glaucoma [8,9,10,11].

Among the different MIGS devices, the ab interno gel Implant XEN^®^ (Allergan, Dublin, Ireland) allows flow of aqueous humor from the anterior chamber to the subconjunctival space [8,9,10,11].

Although many studies have shown the good efficacy/safety profile of XEN45 [12,13,14,15,16,17], the evidence assessing the outcomes of XEN63 is very limited [18,19,20,21] and those trials were done with a previous version of the device during development, which was never commercialized and differs from the current commercially available XEN63 device in many aspects (i.e., needle gauge, injector design, implantation technique, etc.).

**Figure 1:**
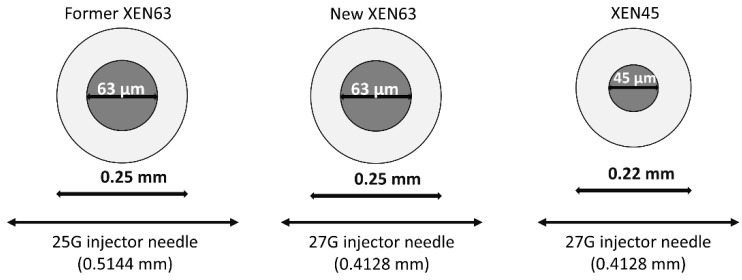
A comparison of the outer and inner diameters of the former XEN63, new XEN63, and XEN45 devices. The former XEN63 device was inserted by using a 25G needle injector (with an outer diameter of 0.5144 mm), while the new XEN63 device is inserted by using a 27G needle injector (with an outer diameter of 0.4128 mm, which is 19.8% smaller). As compared to XEN45 implant, the outer diameter of XEN63 is only 12% greater, while the inner diameter is 1.4 times greater. Since the new XEN63 and the XEN45 devices are inserted by using a 27G injector needle, the side flow with the XEN63 is reduced compared with the XEN45.

The main difference between XEN45 and XEN63 is the bore of the stent [10,11,18,19,20,21]. The new XEN63 device is introduced by using a 27G needle, similarly to the XEN45 stent. Since the outer diameter of XEN63 is greater than that of XEN45, the side flow with the XEN63 is reduced compared with the XEN45 (Figure 1).

The new XEN63 device was developed for decreasing the incision site as compared to the former XEN63 device, and at the same time increasing the aqueous humor flow rate as compared to XEN45, which would provide lower IOPs.

The purpose of this study is to assess the efficacy in terms of IOP lowering and reduction in number of ocular hypotensive drugs of the upcoming new model of XEN63 stent implant. Additionally, the current study also aimed to evaluate the incidence of adverse events in the early postoperative period.

## 2. Materials and Methods

### 2.1. Design

Retrospective, open-label, and bicenter clinical study.

The study protocol was approved by the Ethic Committee of the University of Torino, which waived the need for written informed consent. The study was conducted in accordance with the principles of the Declaration of Helsinki.

### 2.2. Patients

The study was conducted on consecutive OAG patients who underwent a XEN63 implant, either alone or in combination with cataract surgery, between February and June 2020.

All participants were required to meet the following inclusion criteria: age ≥40 years, clinical diagnosis of OAG, and an unmet target IOP despite medical therapy. Patients with narrow-angle glaucoma (unless the surgeon believed that there was sufficient space to safely implant the device), severe conjunctival scars, ocular pemphigoid, phacodonesis, progressive retinal or optic nerve disease of any cause, or history of major ocular surgery (except phacoemulsification) within the previous 6 months were excluded from the study.

### 2.3. Device

In the current study, a MIGS device (Allergan, Irvine, CA, USA) was used. It is composed of porcine gelatin crosslinked with glutaraldehyde. The stent is 6 mm in length, with an outer diameter of 250 μm and an inner diameter of 63 μm.

### 2.4. Surgical Technique

All the surgical procedures were performed, under local anesthesia, by the same two experienced surgeons (AMF and MF).

The XEN implant was placed in the superior nasal quadrant using a standard ab interno technique [16,17,20]. After anesthesia and skin disinfection, conjunctival upper-nasal quadrant was marked 3 mm from the limbus. Before surgery, 0.1 mL of mitomycin C (MMC) 0.02–0.03% was injected intra-tenon in the supero-nasal quadrant.

After injecting a viscoelastic with high cohesivity, the pre-loaded injector needle was inserted at the inferotemporal quadrant through a 1.8 mm corneal paracentesis. An intraoperative goniolens was used to verify placement through the non-pigmented part of the trabecular meshwork. Once the goniolens was removed, the tip was advanced approximately 3 mm through the sclera, and the implant was finally positioned into the subconjunctival space. The position of the implant in the anterior chamber was checked, by gonioscopy, before removing the viscoelastic. In order to confirm the lack of adhesions, sideways movements of the implant were performed until it moved freely under the conjunctiva.

Afterwards, implant function and bleb formation were assessed by constant irrigation with balanced salt solution (BSS). Finally, the corneal incisions were hydrated with BSS.

In eyes that underwent cataract surgery, phacoemulsification was performed using the surgeon’s preferred technique and XEN63 was implanted in all cases after cataract surgery.

Perioperative care included antibiotic therapy 4 times a day for 1 week and anti-inflammatory therapy with steroids 6 times daily, which was slowly tapered over three months.

At baseline, each subject underwent a standard ophthalmic exam, which included a detailed medical history, anterior segment and fundus examination, best corrected visual acuity (BCVA), IOP measurement assessed at 9 am (±1 h) using Goldmann applanation tonometry, and gonioscopy. A computerized visual field (Humphrey visual field analyzer; Carl Zeiss Meditec, Dublin, CA, USA) performed within 6 months before XEN63 implantation was considered as the baseline examination.

Follow-up visits included anterior segment examination, paying special attention to filtering bleb, BCVA, IOP, dilated fundus examination, and the incidence of adverse events.

Topical and systemic IOP-lowering medications were suspended on the day of surgery.

Patients with bleb fibrosis, flat bleb, and/or elevated IOP underwent needling, which was performed in the theater.

Special attention was paid to avoid or delay ocular hypotensive drug reintroduction as much as possible. Before starting any postoperative antiglaucoma medications, surgeons performed either needling or bleb revision. If this approach failed or the patient refused to undergo these procedures, topical hypotensive medication was reintroduced.

### 2.5. Outcomes

Primary endpoints were the IOP at month 3 and the incidence of serious adverse events.

Secondary endpoints included incidence of any adverse event, reduction in number of ocular hypotensive medications from baseline to month 3, proportion of patients achieving an IOP lowering ≥30% and ≥20% without antiglaucoma medications, proportion of patients achieving a final IOP ≤12 mm Hg, ≤14 mm Hg, ≤16 mm Hg, or ≤18 mm Hg without medications, and incidence of non-serious adverse events.

### 2.6. Statistical Analysis

A standard statistical analysis was performed using Prism 9 version 9.0 (GraphPad Software; San Diego, CA, USA).

Although sample size was not calculated before the study, we conducted a post hoc analysis for testing the adequacy of the sample. The post hoc power analyses was determined for an alpha level of 0.05, the study sample size, and the effect size observed in the study [22].

Data are expressed as number (percentage), mean ± standard deviation (SD), or mean (95% confidence interval, CI) as appropriate.

Data were tested for normal distribution using a Shapiro–Wilks test.

Changes in IOP and number of ocular hypotensive medications were performed by means of repeated measures ANOVA and the Greenhouse–Geisser correction test.

The last-observation-carried-forward method was used to impute missing data.

A *p* value of less than 0.05 was considered significant.

## 3. Results

Twenty-three patients met the inclusion/exclusion criteria requirements.

Mean age was 67.8 ± 15.3 years and 15 (65.2%) were women. Table 1 shows the main baseline clinical and demographic characteristics of the study population.

**Figure 2:**
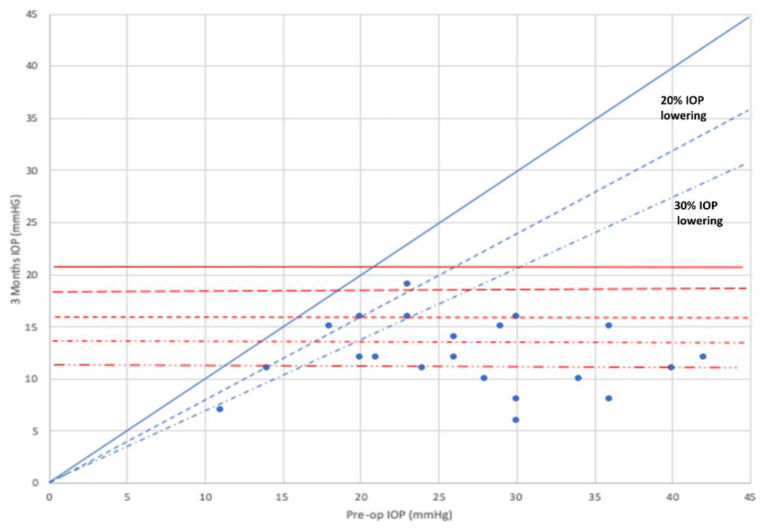
Scatter plot of the intraocular pressure at baseline and month 3. Mean difference −14.8 ± 6.0 mm Hg, 95 Confidence interval −18.4 to −11.2 mm Hg; *p* < 0.0001 (two-tailed paired-samples Student *t* test). The 20% and the 30% lines indicate the level beneath which an IOP reduction of more than 20% or 30%, respectively, compared to baseline value before surgery was reached by the individual cases. IOP: Intraocular pressure.

In the overall study population, baseline IOP was significantly reduced from 27.0 ± 7.8 mm Hg to 12.2 ± 3.4 mm Hg at month 3 (*p* < 0.0001) (Figure 2).

**Figure 3:**
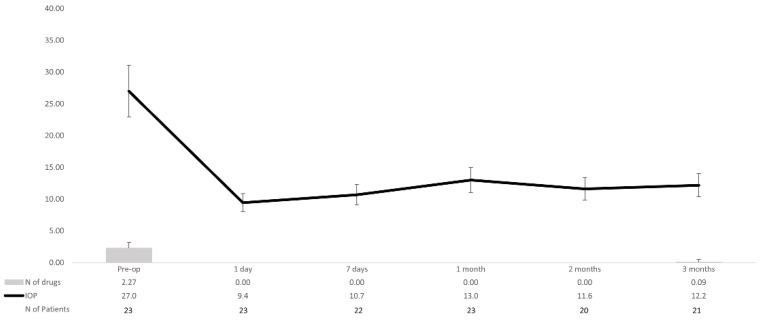
Overview of the mean intraocular pressure (IOP) and number of hypotensive medications over the course of the study follow-up in the overall study population. Vertical bars represent standard deviation. IOP: Intraocular pressure.

Figure 3 shows the mean IOP over the course of the study follow-up.

When compared to baseline, mean (95% confidence interval) IOP lowering was −17.6 (−22.0 to −13.1) mm Hg, *p* < 0.0001; −16.3 (−20.8 to −11.8) mm Hg, *p* < 0.0001; −14.0 (−18.6 to −9.4) mm Hg, *p* < 0.0001; −15.4 (−20.2 to −10.6) mm Hg, *p* < 0.0001; and −14.8 (−20.1 to −9.5) mm Hg, *p* < 0.0001 at day 1, day 7, and months 1, 2, and 3, respectively.

At month-3, mean lowering IOP was 40.8 ± 23.5%, with 14 (60.9%) and 16 (69.6%) eyes achieving an IOP lowering ≥30% and ≥20% without hypotensive medication, respectively.

Mean number of topical ocular hypotensive medications was significantly reduced from 2.27 ± 0.94 drugs at baseline to 0.09 ± 0.42 drugs at month 3 (*p* < 0.0001). At month 3, 22 (95.7%) eyes did not receive any antiglaucoma medication.

BCVA did not change over the course of the study (mean change: 0.1 ± 0.2).

At day-7, 4 (17.4%) eyes showed a ≥2-line worsening in BCVA as compared to baseline, where two of them belonged to the combo group (Table 2).

Regarding safety, four (17.4%) eyes had hypotony (an IOP ≤ 6 mm Hg) at postoperative day one, which was successfully resolved without sequelae and resolved with medical therapy within a month. Three (13.0%) eyes required needling over the course of the study follow-up (mean time for needling 35.6 ± 9.7 days), one eye with mitomycin-c and two with 5-fluorouracil. Only one eye underwent needling due to elevated IOP. Five (21.7%) eyes underwent digital ocular massage. One (4.3%) eye had anterior chamber bleeding during the surgery, one (4.3%) eye had a 1.5 mm hyphema at day 1, and 4 (17.4%) had choroidal detachment (3 at day 7 and 1 at day 15), which was successfully resolved with medical treatment, at the month-1 visit.

## 4. Discussion

Over the past several years there has been growing interest in MIGS devices, mainly due to the need for a safer alternative to traditional surgery.

According to the results of the collaborative initial glaucoma treatment study (CIGTS) [7], trabeculectomy was associated with a fifty percent incidence of early postoperative complications. In the same study, choroidal detachment, anterior chamber bleeding, or anterior chamber flattening had an incidence equal to or greater than 10% [7].

Additionally, the results of the tube versus trabeculectomy study showed that the rate of early postoperative complications (those developed within the first month after surgery) was 37% in the trabeculectomy group [23].

According to the results of the current study, even if they are limited to only 23 eyes and with short follow-up of 3 months, XEN63 provided a better IOP lowering effect than XEN45 [12,13,14,15,16,17,24,25,26,27].

It is important to mention the good hypotensive profile found in our study, with a mean IOP lowering of 40.8 ± 23.5% and 16 (69.6%) eyes achieving an IOP lowering ≥30% without hypotensive medication.

The XEN45 implant has shown a good early and long-term postoperative safety profile, while maintaining good IOP lowering [12,13,14,15,16,17].

When comparing our results with other studies, which reported IOP data of XEN45 stent at month-3, it can be observed that the mean IOP achieved with XEN63 was consistently lower (see Table 3). The same holds true if we examine the differential reduction in pressure [15,26] (Table 3).

With the exception of the Hengerer et al. study [28], the baseline IOP of our study was higher than that reported by other authors [13,15,16,24,25,26,27]. Despite its short-term follow-up, this study points to the fact that XEN 63, is not only able to achieve lower IOP but also that a greater lumen size of the device may benefit patients with a higher baseline pressure.

The relevance of this finding critically depends on whether early post-operative pressures may be predictive of long-term success. We have evidence suggesting that lower IOP in the early postoperative period was associated with successful outcomes in patients undergoing trabeculectomy [29,30]. Moreover, these findings seem to be applicable to the XEN45 device [16,31]. In a previous study conducted by our group [16], week-1 and month-1 postoperative IOP significantly correlated with the final IOP and those eyes with a lower IOP at week 1 had a higher success rate.

Currently available scientific evidence evaluating the efficacy and safety of the XEN63 implant is very limited and information about the former device was never commercially available [18,19,20,21]. Although in general terms the results of these studies have shown good efficacy and safety profile of the former device, due to differences in the surgical technique and the device, it is difficult to compare our results with those of the previous XEN63 studies.

The main difference between the former XEN63 device and the new one is the surgical technique. While the former XEN63 stent was implanted through a 2.2 mm peripheral corneal incision with a 25G injector needle, the new XEN63 device is implanted through a 1.8 mm corneal paracentesis by using a 27G injector needle (see Figure 1). This new surgical approach reduces the side flow of the new XEN63 as compared with the former one. Additionally, previous studies were performed without MMC [18,19,20,21].

Regarding safety, the most commonly reported adverse event was choroidal detachment (4 eyes), which was successfully resolved without treatment at the month-1 visit.

In this study, four (17.4%) eyes had an IOP ≤ 6 mm Hg at postoperative day one, but they were resolved without consequences.

On this subject, Lenzhofer et al. [19], using the former XEN63 device, reported that 3 (4.7%) eyes required some intervention (between surgery and end of the study) due to low IOP. Additionally, Lavin-Dapena et al. [21], also evaluating the former XEN63 device, found hypotony (similar criterion than ours) in 3 (27.3%) eyes at day 1. However, it should be noted that both studies did not use MMC, which in theory might reduce the incidence of hypotony.

This brings us to the question of whether the better IOP lowering effect obtained with XEN63 would theoretically be associated with a greater risk of hypotony.

Despite the greater inner diameter of XEN63, the incidence of hypotony was not significantly different than that observed with XEN45 [12,13,14,15,16,17]. This may be because the resistance is determined by the subconjunctival bleb [20]. Another explanation may be related to our surgical technique. Since the needle caliper used to make the track through the sclera is smaller than the previous one, the risk of peritubular filtration should not be greater than with XEN45.

Avoidance of hypotony in the early post-operative phase following glaucoma drainage device surgery is paramount if serious complications are to be avoided. Hypotony in the early postoperative period is a common and significant complication that has been associated with delayed visual recovery following trabeculectomy [32,33]. The reason why early post-operative complications are lower than in trabeculectomy may be due to the fact that the pre-determined lumen allows for a much more controlled outflow as compared to the traditional filtration surgery. Although restriction of outflow using different suturing techniques can improve the safety profile and reduce the rate of early complications observed with trabeculectomy, this carries the disadvantage of manipulation of the sutures in the post-operative period [34].

In this study, early hypotony was not related to ocular complications or visual acuity loss. In the overall study sample, mean visual acuity did not change over the course of the study. Although at day-7, 4 (17.4%) eyes had a ≥2-line worsening in BCVA as compared to baseline, two eyes recovered within a month.

Moreover, it should be highlighted that at month 3, eight (34.8%) eyes showed a ≥2-line improvement in BCVA as compared to baseline.

Unfortunately, as far as we know, visual acuity changes have not been reported in detail in previous studies, beyond its relationship with hypotonic maculopathy. This issue makes it extremely difficult to compare our results with other studies.

Vision loss associated with hypotony can be bothersome especially for one-eyed patients. Studies comparing the different kinds of glaucoma treatment using quality of life as an outcome are rare. However, patients that have undergone trabeculectomy have reported a worsening in quality of life in the early postoperative stage, which was directly linked to the local effects of the surgery [35].

Regarding needling, in the present study, 3 (13.0%) eyes underwent post-operative needling, which was a low rate compared to that reported in previous XEN45 papers [12,13,14,15,16,17]. Moreover, it should be noted that in 2 out of these 3 cases, needling was performed as a preventive measure and not because of a frank elevation of IOP.

The current study has several limitations that should be taken into consideration when assessing its results. The first one is its retrospective design. Potential bias and confounding factors are inherent of retrospective studies. Nevertheless, selection of strict inclusion/exclusion criteria tried to minimize their impact. The second limitation is its limited follow-up time. Nevertheless, the assessment of short-term clinical outcomes may be useful, since early postoperative IOP seems to be associated with the success of the procedure. The third limitation is the lack of sample size calculation before starting the study. However, according to the results of the study, the power for detecting mean IOP lowering and ocular hypotensive drug reduction, between baseline and month 3, was 99% for each. Finally, the last limitation was the lack of a control group. It would have been interesting to conduct a head-to-head comparison, preferably a randomized clinical trial, between XEN45 and XEN63.

## 5. Conclusions

The results of the current study clearly suggested that XEN63 was an effective and safe surgical procedure in OAG patients. XEN63 significantly lowered IOP and reduced the number of antiglaucoma medications, with a good safety profile. Its limited incidence of hypotony, in combination with a better understanding of the use of MMC (both in terms of concentration and area of injection), may allow physicians to treat more advanced patients and to obtained a lower target-IOP in the long-term.

Further research is needed to assess its long-term clinical outcomes, as well as to identify potential factors associated with clinical success.

## Figures and Tables

**Table 1:** Main baseline clinical and demographic characteristics of the study sample.

Variable	Overall (*n* = 23)	XEN 63 (*n* = 20)	Phaco + XEN63 (*n* = 3)
Age, years			
Mean ± SD	67.8 ± 15.3	67.3 ± 15.9	71.3 ± 12.4
Sex, *n* (%)			
Women Men	15 (65.2) 8 (34.8)	14 (70.0) 6 (30.0)	1 (33.3) 2 (66.7)
Type of glaucoma			
POAG Uveitic PXG PACG Traumatic Missing information	14 (60.9) 4 (17.4) 1 (4.3) 1 (4.3) 1 (4.3) 2 (8.7)	12 (60.0) 3 (15.0) 1 (5.0) 1 (5.0) 1 (5.0) 2 (10.0)	2 (66.7) 1 (33.3) 0 (0.0) 0 (0.0) 0 (0.0) 0 (0.0)
Previous laser, *n* (%)			
No SLT Nd:YAG Iridotomy	21 (91.3) 1 (4.3) 1 (4.3)	18 (90.0) 1 (5.0) 1 (5.0)	3 (100.0) 0 (0.0) 0 (0.0)
Previous surgery *, *n* (%)			
None Cataract Refractive (laser) Trabecular MIG Subconjunctival MIG	2 (8.7) 14 (60.9) 3 (13.0) 2 (8.7) 2 (8.7)	0 (0.0) 14 (70.0) 3 (15.0) 2 (10.0) 2 (10.0)	3 (100.0) 0 (0.0) 0 (0.0) 0 (0.0) 0 (0.0)
BCVA, ETDRS			
Mean ± SD	0.49 ± 0.26	0.54 ± 0.24	0.23 ± 0.15
ECC			
Mean ± SD	2217.9 ± 343.1	2223.4 ± 297.0	2161.0 ± 563.8
MD, dB			
Mean ± SD	−17.03 ± 9.96	−16.71 ± 10.51	−18.23 ± 9.44
PSD, dB			
Mean ± SD	7.00 ± 2.28	7.10 ± 3.08	6.66 ± 3.2
NTOHM			
Mean ± SD	2.27 ± 0.94	2.26 ± 0.99	2.33 ± 0.58
IOP, mm Hg			
Mean ± SD	27.0 ± 7.8	26.5 ± 8.2	30.3 ± 3.2

* Patients may have undergone more than one procedure. Abbreviations: Phaco: Phacoemulsification; SD: Standard deviation; POAG: Primary open-angle glaucoma; PXG: Pseudoexfolitive glaucoma; PACG: Primary angle-closure glaucoma; SLT: Selective laser trabeculoplasty; YAG: Neodymium-doped Yttrium Aluminium Garnet; MIG: Minimally invasive glaucoma device; BCVA: Best corrected visual acuity; ETDRS: Early treatment diabetic retinopathy study; ECC: Endothelial cell count; MD: Mean defect; PSD: Pattern standard deviation; NTOHM: Number of topical ocular hypotensive medications; IOP: Intraocular pressure.

**Table 2:** Overview of the number (%) of patients who experienced changes in best corrected visual acuity throughout the study.

	Day 1	Day 7	Month 1	Month 3
Worse ≥2 lines, *n* (%)	4 (17.4)	4 (17.4)	2 (8.7)	2 (8.7)
Worse ≥1 line, *n* (%)	6 (26.1)	7 (30.4)	3 (13.0)	3 (13.0)
Unchanged, *n* (%)	9 (39.1)	6 (26.1)	8 (34.8)	6 (26.1)
Improvement ≥1 line, *n* (%)	0 (0.0)	2 (8.7)	3 (13.0)	2 (8.7)
Improvement ≥2 lines, *n* (%)	4 (17.4)	4 (17.4)	7 (30.4)	8 (34.8)

**Table 3:** Overview of the intraocular pressure (IOP) and number of hypotensive medications in eyes that underwent XEN45 implant surgery in different studies at month-3.

Study	MMC	Type of Glaucoma	Baseline IOP, mm Hg	M3 IOP, mm Hg	IOP Lowering (%)	IOP Lowering, mm Hg	Mean Preoperative Medications	Mean Postoperative Medications at M3	Needling Rates at the End of the Study, *n* (%)
Reitsamer et al. [13]	10–80 µg/mL ^1^	POAG	21.4 (3.6) *	15.7 ^†^	−25.0 ^†^	N.A.	2.7 (0.9)	0.5 (0.9)	83 (41.1)
Marcos-Parra et al. [15]	10 µg/mL	OAG ^3^	19.1 (5.4) *	N.A.	N.A.	−6.1 (−9.9 to −0.1) **	2.5 (0.8)	N.A.	13 (20.0)
Fea et al. [16]	20 µg/mL	OAG ^3^	23.9 (7.6) *	15.1 ^†^	N.A.	N.A.	3.0 (1.0)	0.4 ^†^	79 (46.2)
Grover et al. [24]	20 µg/mL ^2^	Refractory OAG ^3^	25.1 (3.7) *	16.6 (5.5) *	−32.7 ^†^	−8.5 ^†^	3.5 (1.0)	0.5 ^†^	21 (32.3)
Ibáñez-Muñoz et al. [25]	10 µg/mL	OAG ^3^	22.8 (20.8 to 24.7) **	16.4 (14.3 to 18.5) **	N.A.	N.A.	3.4 (0.8)	N.A.	19 (26.0)
Laborda-Guirao et al. [26]	20 µg/mL	OAG ^3^	21.0 (5.2) *	14.5 (13.6 to 15.4) **	N.A.	−6.7 (−8.8 to −4.6)	2.8 (2.7 to 3.0) **	N.A.	7 (8.8)
Theilig et al. [27]	10 µg/mL	POAG	24.5 (6.7) *	16.8 (6.3)	N.A.	N.A.	3.0 (1.1) *	1.1 (1.4) *	42 (42.0)
Hengerer et al. [28]	10 µg/mL	OAG ^4^	32.2 (9.1) *	14.6 ^†^	N.A.	N.A.	3.1 (1.0) *	−2.7 (1.2) ^‡^	67 (27.7) ***
Current study	20–30 µg/mL ^1^	OAG ^3^	27.0 (7.8) *	12.2 (3.4) *	−40.8 (23.5) *	−14.8 (−20.1 to −9.5) **	2.3 (0.9) *	0.1 (0.4) *	3 (13.0)

* Mean (Standard deviation); ** Mean (95% confidence interval); ^†^ Data about standard deviation was not provided; ^‡^ Mean reduction from baseline; *** All the needling procedures were done between week 1 and month 3; Abbreviations: MMC: Mitomycin C; IOP: Intraocular pressure; M: Month; POAG: Primary open-angle glaucoma, OAG: Open-angle glaucoma; NA: Not available. ^1^ MMC dose at the surgeon’s discretion (2 patients received 5-fluorouracil); ^2^ Sponges saturated with MMC; ^3^ It includes primary and secondary open-angle glaucoma; ^4^ Besides open-angle glaucoma patients, it included patients with uveitic glaucoma, angle closure glaucoma, and neovascular glaucoma.

## Data Availability

The data presented in this study are available on reasonable request from the corresponding author.
